# Extended Scalp Reconstruction With a Free Supraclavicular Graft: A Case Report

**DOI:** 10.7759/cureus.34324

**Published:** 2023-01-29

**Authors:** Chrysoula Vardaxi, Paraskevi Karamitsou, James Philip Skliris, Spyridon Gougousis, Alexandros Poutoglidis

**Affiliations:** 1 Otorhinolaryngology-Head and Neck Surgery, George Papanikolaou General Hospital, Thessaloniki, GRC; 2 Pathology, George Papanikolaou General Hospital, Thessaloniki, GRC

**Keywords:** donor site, tissue transfer, facial plastic surgery, skin reconstruction, supraclavicular graft

## Abstract

Skin restoration after tumor excision, trauma, or burns may be achieved with full or split-thickness skin grafts or local flaps. The success rate of a skin graft depends on several independent factors. The supraclavicular region is considered a reliable donor site for head and neck skin defects because of its easy access. We present a case of a supraclavicular skin graft harvested to cover a skin deficit after the excision of a squamous cell carcinoma of the scalp. The postoperative course was uneventful regarding graft survival, the healing procedure, and the cosmetic outcome.

## Introduction

Using skin flaps and grafts is a well-established and reliable method for skin reconstruction in plastic surgery. Although the surgical technique has been used for several decades, the basic principles remain similar [1]. Free grafts are harvested from distant areas, and their survival depends on the angiogenesis in the recipient site. Grafts are either full thickness containing all skin layers above the subcutaneous tissue, or split-thickness consisting only of the epidermis and a part of the dermis [2]. Thorough consideration of skin color, thickness, and consistency is required to decide the most suitable donor site [1,3]. The most commonly used free grafts for head and neck reconstruction are derived from the major pectoralis muscle, the forearm, and the face [3,4]. The supraclavicular area is also a reliable donor option to cover large head and neck defects due to its similarity in color, thickness, and flexibility [4]. We report a case of a squamous cell carcinoma of the scalp, which was treated successfully with tumor excision followed by skin reconstruction with a supraclavicular graft.

## Case presentation

**Figure 1:**
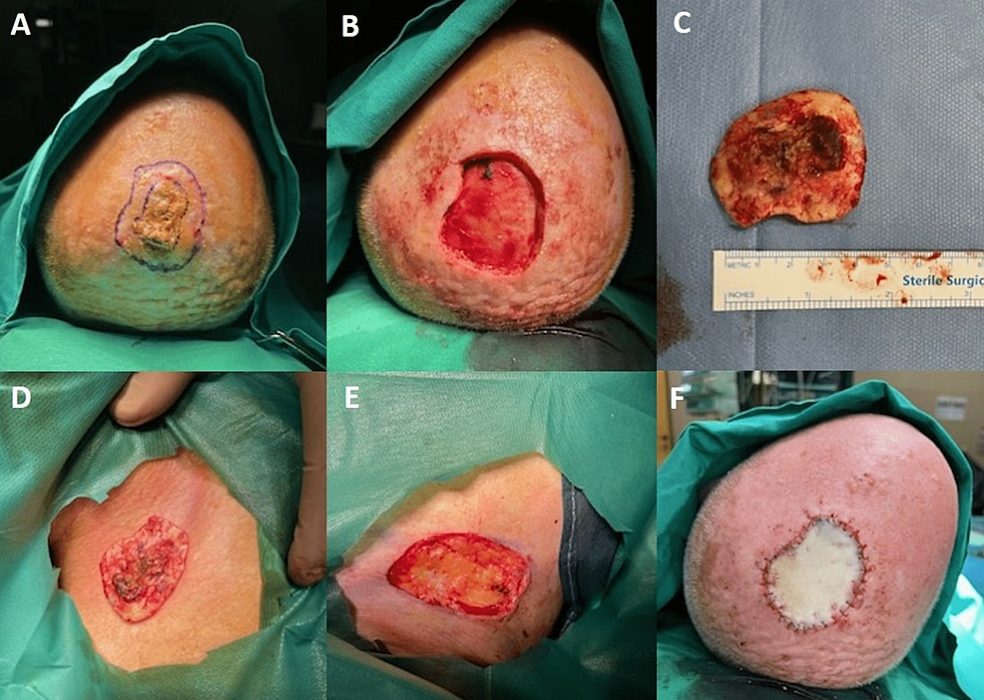
Description of the surgical procedure A: Lesion's removal with macroscopically negative surgical margins of 1 cm; B: Depth of excision up to the periosteum of the skull; C: The surgical specimen; D: Measurement of the size of the required free skin graft from the supraclavicular area; E: Donor site (supraclavicular area); F: Graft's incorporation with continuous interlocking absorbable sutures

A 70-year-old male presented to our otolaryngology outpatient clinic with the chief complaint of a progressively grown skin lesion on the scalp. The lesion first appeared one year ago. His medical history included hypertension, hypothyroidism, and coronary disease. A thorough physical examination revealed a well-defined, crusted lesion on the scalp of approximately 3.8 cm x 3 cm. Surgical excision was performed under general anesthesia. The lesion was removed with macroscopically negative surgical margins of 1 cm (Figure 1 A). The depth of the excision was up to the periosteum of the skull (Figure 1 B). Once the lesion was removed, the surgical specimen was used to measure the size of the required free skin graft (Figure 1 C and D). The loose skin from the right supraclavicular area was recruited to cover the skin defect in the scalp (Figure 1 E). Thus, a full-thickness graft was created for scalp reconstruction. The donor site was primarily closed with simple interrupted sutures. The graft was incorporated with continuous interlocking absorbable sutures (Figure 1 F).

**Figure 2:**
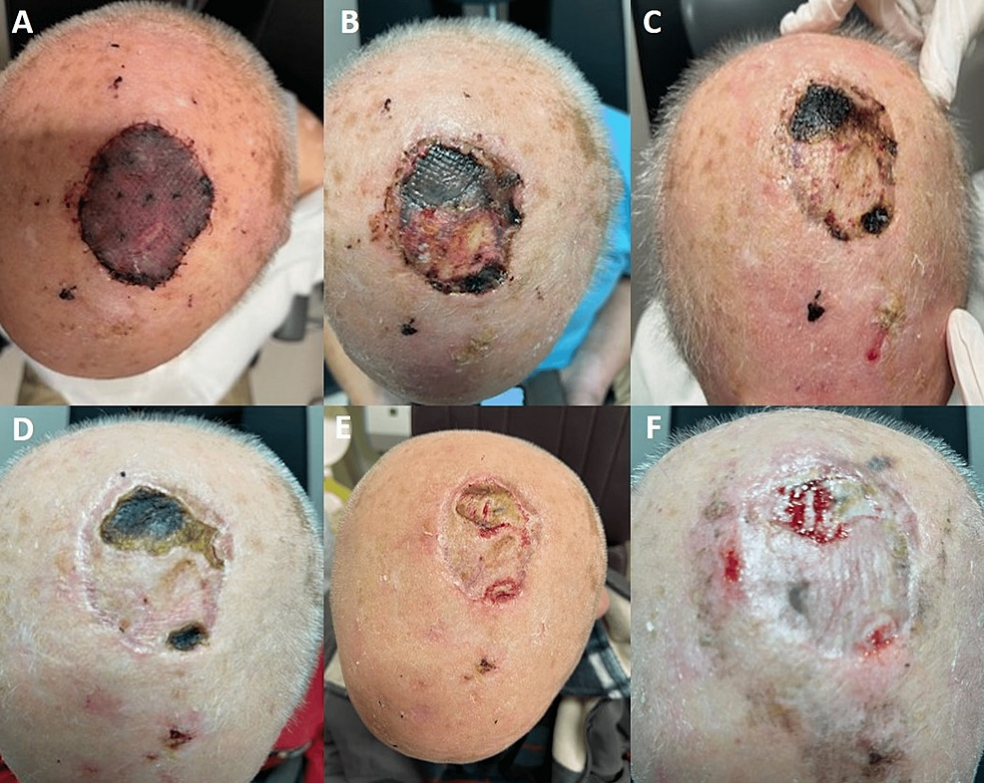
The timeline of the postoperative course and the healing process A: Third postoperative day; B: One week after the surgery; C: Two weeks after the surgery; D: Three weeks after the surgery; E: Four weeks after the surgery; F: Eight weeks after the surgery

At the surface of the graft, numerous buttonholes were created to prevent the formation of a postoperative hematoma. A tight dressing with bolster dressing was recruited to apply adequate pressure on the skin graft. The timeline of the postoperative course and the healing process is demonstrated in Figure 2. A month after the reconstruction, we performed debridement of the crusts (in places where the graft was not integrated properly) at the posterior part of the graft to promote healing by secondary intention.

**Figure 3:**
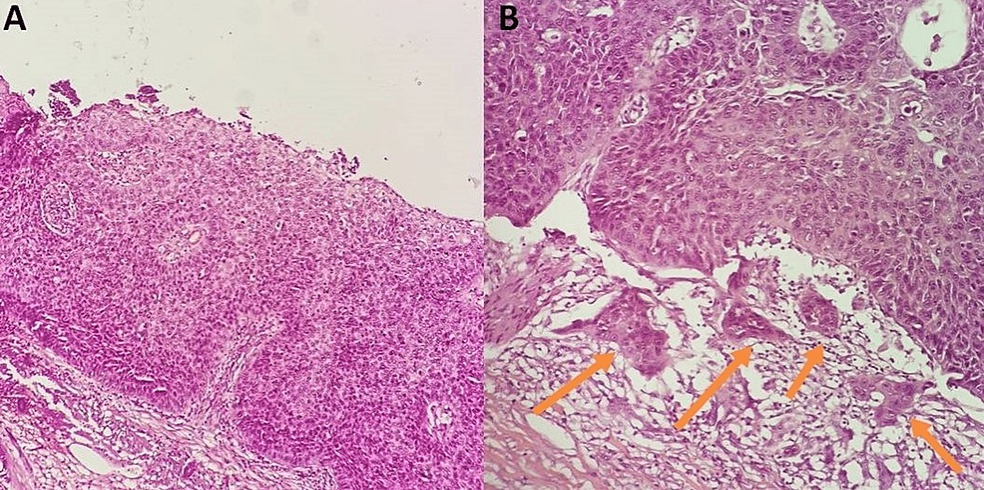
Histology report A: Well-differentiated squamous cell carcinoma with full thickness atypical keratinocytes, hematoxylin and eosin staining (H&E), 10x; B: Focal papillary dermis invasion by malignant cells (arrows), H&E staining, 20x

The concomitant histology report revealed the presence of a squamous cell carcinoma (SCC) of the skin with wide negative surgical margins (Figure 3).

## Discussion

Squamous cell carcinoma is the most common neoplasm of the head and neck area [5]. Excision of skin lesions may require restoration either with local flaps or skin grafts to achieve a decent cosmetic and functional outcome [6]. The most common donor areas to cover skin defects of the head and neck area are the posterior auricular skin, the preauricular skin, the nasolabial fold, the forearm, the thigh, the major pectoralis muscle, and the abdominal area [1,3,7,8]. The supraclavicular area is gaining ground in tissue reconstruction and restoration of small and large deficits [9]. According to some authors, it offers a lower failure rate due to its thinner size [3].

The success of a skin graft surgery is strongly related to the choice of the most appropriate donor site. The recipient site's specific characteristics should be considered to make the proper decision [1,3]. A split-thickness graft could be expanded in case of a large deficit, offering better adherence on the surgical bed but a less favorable aesthetic outcome [1]. Fixation of the graft is paramount to promote the angiogenesis between the graft and the recipient area. It also prevents the formation of a hematoma or a seroma beneath the graft. Applying a tight dressing, such as the tie-over technique, capable of providing pressure on the graft, is usually recommended [1]. Interestingly, Dhillon et al. compared the postoperative outcome in cases where a tie-over dressing was applied to patients with a total absence of dressing, concluding that the fixation of a dressing makes no significant difference in the infection rate or the successful healing of the graft [9].

According to the literature, the supraclavicular artery island flap is a desirable alternative for reconstruction using microsurgical techniques. The features and the use of the supraclavicular graft and other free skin grafts are not inferior to the local flaps [10]. Both options provide similar complication rates with no statistical difference in skin loss or necrosis [4,10].

## Conclusions

Tissue transfer is a widely known technique for skin reconstruction. Various sites could be potentially used as donor or recipient areas. The supraclavicular graft is a reliable, safe, and cosmetically acceptable choice for reconstructing head and neck defects.
